# TLR9–IL-2 axis exacerbates allergic asthma by preventing IL-17A hyperproduction

**DOI:** 10.1038/s41598-020-75153-y

**Published:** 2020-10-22

**Authors:** Yusuke Murakami, Takashi Ishii, Hiroki Nunokawa, Keigo Kurata, Tomoya Narita, Naomi Yamashita

**Affiliations:** 1 Faculty of Pharmacy, Department of Pharmaceutical Sciences, Musashino University, Nishitokyo-shi, Tokyo 202-8585 Japan; 2 ITEA, Inc., Tokyo, Japan

**Keywords:** Immunology, Diseases

## Abstract

Allergic asthma is one of most famous allergic diseases, which develops lung and airway inflammation. Recent studies have revealed the relationship between the pathology of allergic asthma and the increase of host-derived DNA in inflamed lung, but the role of the DNA-recognizing innate immune receptor for the inflammation is unknown well. Here we investigated the role of Toll-Like Receptor 9 in the pathogenesis of allergic asthma without synthesized CpG-ODNs. To examine that, we analyzed the pathology and immunology of house-dust-mite (HDM)-induced allergic asthma in *Tlr9*^*–/–*^ mice and TLR9-inhibitory-antibody-treated mice. In *Tlr9*^*–/–*^ mice, airway hyperresponsiveness (AHR) and the number of eosinophils decreased, and production of the Th2 cytokines IL-13, IL-5, and IL-4 was suppressed, compared with in wild-type mice. Interestingly, unlike Th2 cytokine production, IL-17A production was increased in *Tlr9*^*–/–*^ mice. Furthermore, production of IL-2, which decreases IL-17A production, was reduced in *Tlr9*^*–/–*^ mice. Blockade of TLR9 by treatment with TLR9-inhibitory-antibody, NaR9, effectively suppressed the development of allergic asthma pathology. IL-17A production in NaR9-treated mice was enhanced, which is comparable to *Tlr9*^*-/-*^ mice. These results suggest that the TLR9–IL-2 axis plays an important role in Th2 inflammation by modulating IL-17A production in HDM-induced allergic asthma and that targeting of TLR9 might be a novel therapeutic method for allergic asthma.

## Introduction

Allergic asthma is one of allergic disease in which T and B lymphocytes, eosinophils, basophils, mast cells, and recently discovered type 2 innate lymphocytes (ILC2) cooperate to form the pathology. The onset of this disease is due to inhalation of various allergens including components from house dust mite (HDM), molds, and pets^1^. There are 300 million asthmatics worldwide, and the number of patients is increasing year by year as the abundance of allergens increases because of global warming and changes in the living environment^2,3^. The way to control the onset is mainly by inhalation of steroids, but some patients—about 10%—are resistant to steroid preparations. New, alternative treatments therefore need to be developed^4^.

Vigorous research has been conducted into the pathogenesis of allergic asthma. Production of Th2 cytokines such as IL-13, IL-5, and IL-4 by T cells, as well as IgE production by activated B cells, is very important in the development of pathology^1^. Moreover, recent studies have reported the discovery of type 2 innate lymphoid cells^1,5,6^, which also produce Th2 cytokines, but without antigen presentation. In addition to these cell subsets, various cells expressing innate immune receptors (e.g., Toll-like receptors, TLRs) are involved in allergic asthma^7–10^. For example, the endotoxin sensor Toll-like receptor 4 (TLR4) exacerbates the pathogenesis of HDM-induced allergic asthma^7–9^ and its antagonists suppress allergic asthma^10^. Moreover, TLR4 ligand promotes the pathogenesis of OVA-induced allergic asthma in TNF-dependent manner^11^. On the other hand, the modifications through TLRs influence pathophysiology of asthma both in progressive and suppressive ways. Bacterial flagellin sensor TLR5 also complicates the pathology of asthma model^12^. However, TLR7 ligand inhibits the pathogenesis of allergic asthma^13,14^. These findings suggest that the activation of TLR is important for the pathogenesis of HDM-induced allergic asthma, and that more detailed analyses, including of other TLRs, are needed.

Toll-like receptor 9 (TLR9) is localized to endosomes and lysosomes, and contributes to host defenses, mainly by recognizing CpG DNA from pathogens, such as viruses and bacteria, and activating the immune system^15–17^. Th2 immune response have been reported to be suppressed by CpG-oligonucleotides (ODNs), which are TLR9 agonists^18,19^. Clinical trials using CpG-DNA have been conducted to treat allergic asthma patients. In the trials, a certain improvement effect has been reported^20^. In addition, IL-10-producing lung interstitial macrophages and type-I-interferon-producing plasmacytoid dendritic cells (DCs), which express TLR9, contribute the effect of treatment with ODNs in murine allergic asthma^18,19,21^.

However, not only in the case of pathogen invasion and exogenous ligands, but also in non-infectious inflammatory diseases, self-derived DNA (self-DNA) activates TLR9 to exacerbate pathology^22,23^. In autoimmune diseases such as systemic lupus erythematosus and psoriasis, autoantibodies against self-DNA form a complex with it and protect it from degradation by nucleolytic enzymes; thereafter, the antibody–self DNA complexes reach intracellular endosomes–lysosomes and activate TLR9^24^. This activation contributes to the development of pathology in a variety of autoimmune diseases^22,23^. However, there are few reports on the interaction between allergic asthma and HDM-derived DNA or self-DNA associated with inflamed tissue^25–27^. HDM extract-derived double-stranded RNA stimulates TLR3 and induce interferons, which negatively modulate allergic asthma inflammation^27^. The abundance of neutrophil DNA, which is released extracellularly by uncontrolled neutrophil extracellular traps formation (NETosis), increases in the rhinovirus-infected- and HDM-sensitized lung, and that host genome DNA released from cells damaged by HDM inhalation aggravates the pathology of allergic asthma^25,26^. We therefore need to elucidate how HDM-derived DNA or self-DNA is recognized by TLR9 and is involved in the pathology of HDM-induced allergic asthma.

Recently, we have established monoclonal antibodies against nucleic-acid-recognizing TLR3, TLR7, and TLR9. Each of these antibodies functions to positively or negatively regulate the TLR response and can be expected to be useful as a therapeutic drug for various diseases^28–32^. For example, the TLR7 mAb A94B10 has been reported to rescue the lethal systemic inflammatory response in mice with a *D34A* mutation in the *Unc93-homolog b1* gene^29,33^. The TLR9 mAb NaR9 rescues mice from lethal liver inflammation induced by co-stimulation with CpG-ODN and β-(+)-galactosamine^30^.

Here, we induced HDM allergic asthma in *Tlr9*^*−/−*^ mice and analyzed their pathology in comparison with that of wild-type (WT) mice. In addition, we tried to verify the therapeutic effect of TLR9-inhibitory antibodies in HDM-induced allergic asthma. Our aim was to elucidate the role of the TLR9 response in allergic asthma induced by HDM.

## Results

### ***Tlr9***^***−/−***^ mice have decreased AHR and numbers of inflammatory cells infiltrating the lung

**Figure 1:**
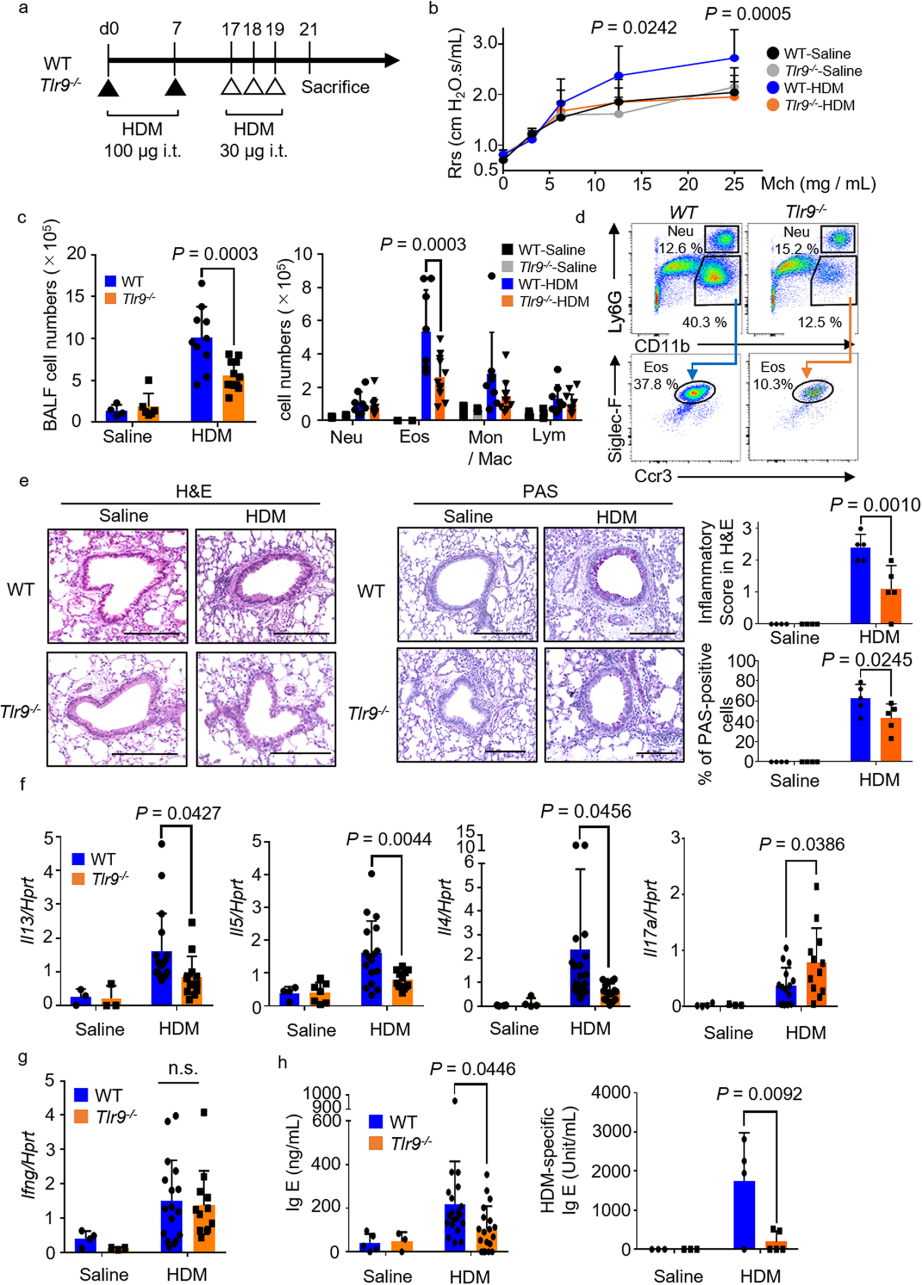
House dust mite (HDM)-induced allergic asthma is attenuated in *Tlr9*^*−/−*^ mice. (**a**) Protocol used for HDM sensitization. (**b**) Airway hyperresponsiveness in response to increasing doses of methacholine (Mch) was measured in wild-type (WT) or *Tlr9*^*−/−*^ mice. (**c**) Numbers of bronchoalveolar lavage fluid (BALF) cells (left panel) or neutrophils (Neu), eosinophils (Eos), monocytes/macrophages (Mon/Mac), and lymphocytes (Lym) (right panel) from the mice indicated were counted. (**d**) Neutrophils (CD11b^+^, Ly6G^+^) and eosinophils (CD11b^+^, Ly6G^−^, Siglec-F^+^, CCR3^+^) in BALF were analyzed by flow cytometry. (**e**) Representative images of hematoxylin and eosin (H&E) and periodic acid–Schiff (PAS)-stained histologic sections of the lungs of mice. Scale bars, 200 μm. The inflammatory score (0 to 3) and the percentage of PAS-positive cells were statistically analyzed. (**f**) Expression levels of the genes indicated were measured in the lungs from HDM-sensitized mice. (**g**)* Ifng*/*Hprt* mRNA was measured in lungs inflamed by HDM sensitization. (**h**) Serum total IgE and HDM-specific IgE were measured by ELISA in the mice indicated. Data are presented as means ± SD of three or four independent experiments (n = 3 to 5 each). *n.s.* not significant, *WT* wild type, *i.t.* intratracheally, *Rrs* respiratory resistance.

First, to evaluate the role of TLR9 in HDM-induced allergic asthma, we induced this asthma in WT mice and *Tlr9*^*−/−*^ mice (Fig. 1a). Measurement of AHR by methacholine administration showed a significant decrease in HDM-treated *Tlr9*^*−/−*^ mice compared with HDM-treated WT mice (Fig. 1b). In addition, the number of cells in the BALF, including eosinophils, was significantly lower in HDM-treated *Tlr9*^*−/−*^ mice than in HDM-treated WT mice (Fig. 1c). Eosinophil (CD11b^+^, Siglec-F^+^, CCR3^+^, Ly6G^-^) loss in *Tlr9*^*−/−*^ mice was confirmed by flow cytometry (Fig. 1d). Next, we performed HE and PAS staining to analyze pathology in the lungs (Fig. 1e). In HDM-treated WT mice, cell infiltration around the bronchi was observed upon HE staining, whereas it was markedly reduced in HDM-treated *Tlr9*^*−/−*^ mice. PAS staining showed that the abundance of PAS-positive cells was lower among the bronchial epithelial cells of HDM-treated *Tlr9*^*−/−*^ mice than among those of HDM-treated WT mice. These results suggested that the TLR9 response exacerbated HDM-induced allergic asthma.

### Th2 cytokine and IgE production, but not IFN-γ production, are altered in ***Tlr9***^***−/−***^ mice during allergic asthma

Production of Th2 cytokines is strongly involved in the development of allergic pathologies^1^. Therefore, we recovered mRNA from the lungs of WT and *Tlr9*^*−/−*^ mice and analyzed the levels of Th2 cytokine transcripts. The mRNA levels of *Il-13*, *Il-5*, and *Il-4* were significantly lower in HDM-treated *Tlr9*^*−/−*^ mice than in HDM-treated WT mice. In contrast, *Il17a* mRNA expression in *Tlr9*^*−/−*^ mice was significantly higher than that in WT mice (Fig. 1f). However, IFN-γ transcript levels did not differ between HDM-treated WT and *Tlr9*^*−/−*^ mice (Fig. 1g). This result suggested that the TLR9 response was involved in Th2 cell activation, but not in IFN-γ production. Finally, we also measured serum IgE production, which is the result of IL-4 mediated class switching in B-cells and is therefore a hallmark of allergic disease^1^. HDM-treated *Tlr9*^*−/−*^ mice had significantly lower Total IgE and HDM-specific IgE production than those of HDM-treated WT mice (Fig. 1h).

### TLR9 response to HDM-DNA is partly involved in total DC immune response to HDM

Because we found that TLR9 involved in the pathogenesis of HDM-induced asthma (Fig. 1), we investigated whether the DNA ligand involved was derived from HDM (Supplementary Fig. 1). Because HDM extract contains LPS (a TLR4 ligand), we also used *Tlr4*^*−/−*^ mice. First, BM-derived cDCs were induced from the bone marrow of WT mice, *Tlr4*^*−/−*^ mice, and *Tlr9*^*−/−*^ mice, and the cells were then stimulated with HDM, LPS, CpG-ODN, or Pam3csk4 (a TLR2 ligand). Pam3csk4 did not change RANTES production in WT, *Tlr4*^*−/−*^ and TLR9KO cDCs. At low concentrations of HDM (10 μg/mL), CCL5 production was significantly lower in *Tlr9*^*−/−*^ cDCs than in WT cDCs, and it was completely abolished in *Tlr4*^*−/−*^ cDCs. At high concentrations of HDM (30 μg/mL), there was no difference in CCL5 production between *Tlr9*^*−/−*^ cDCs and WT cDCs. Next, we examined whether HDM-DNA was involved in the STING response, because STING is cytoplasmic DNA sensor. *Sting*^*−/−*^ cDCs had significantly greater CCL5 production than WT cDCs in response to HDM stimulation (Supplementary Fig. 1b). Because the TLR4 response to LPS has a possibility to mask the TLR9 response, we used HDM-DNA digested or not with DNase I (Supplementary Fig. 1c) to treat *Tlr4*^*−/−*^ cDCs. CCL5 production was significantly lower upon stimulation with DNase I-treated HDM, whereas IL-12 p40 production was not affected by DNase I treatment (Supplementary Fig. 1d). Both CCL5 and IL-12 p40 production did not decrease on stimulation with DNase I-treated loxoribine (a TLR7 ligand). From these results, it was found that TLR9 is partially involved in *Tlr4*^*−/−*^ cDCs responses to HDM-derived DNA.

### TLR9 is expressed on B cells and MHC class II^+^ DCs in the lung

**Figure 2:**
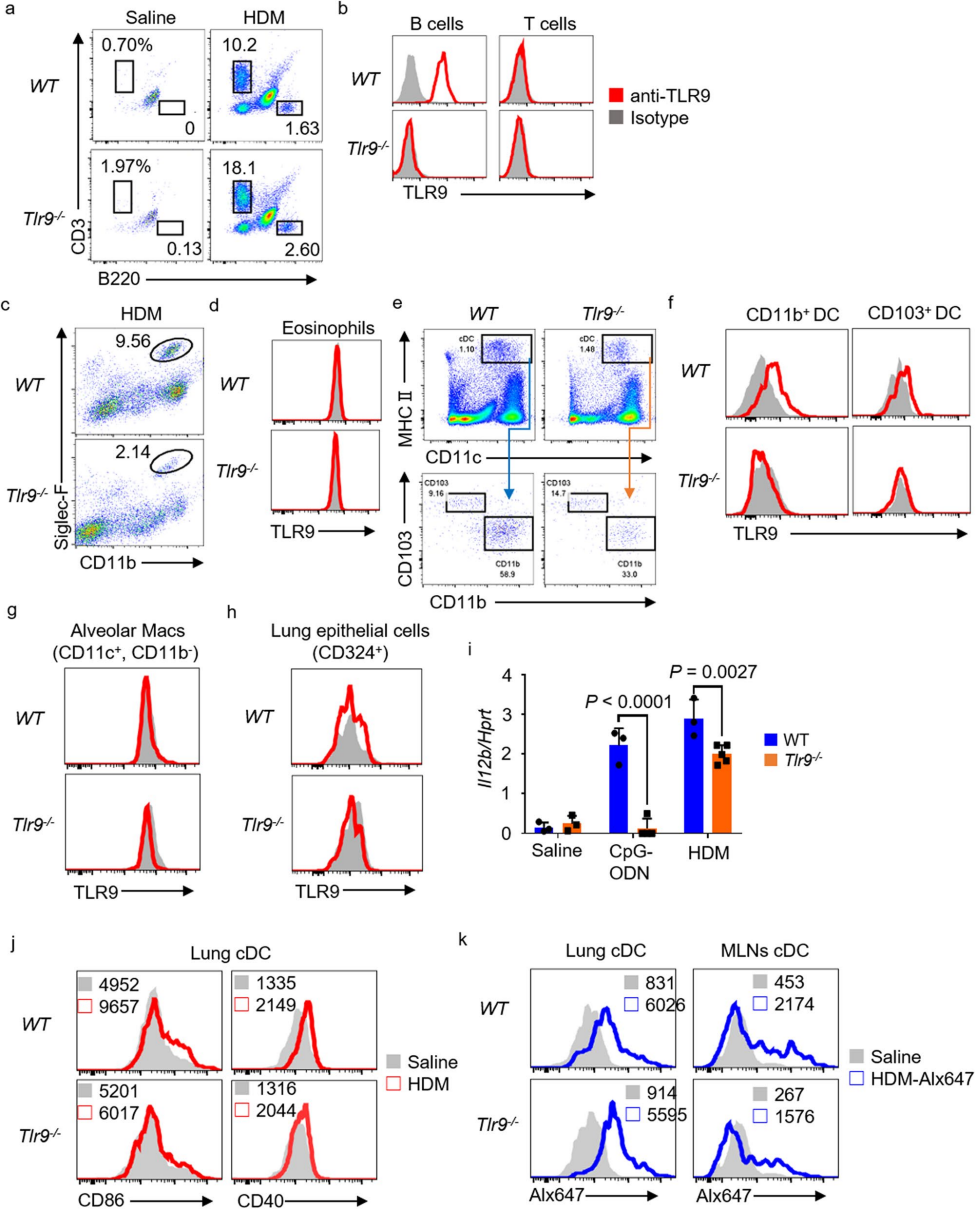
Toll-like receptor 9 (TLR9) is expressed in B cells and dendritic cells (DCs) that infiltrate the inflamed lung. (**a**) Lymphocytes in bronchoalveolar lavage fluid (BALF) were analyzed by flow cytometry for the markers indicated. The percentages of each cell were shown. (**b**) TLR9 expression was examined in B cells and T cells of BALF from HDM-sensitized mice. (**c**) Eosinophils were analyzed by flow cytometry for the markers indicated. (**d**) TLR9 expression was examined on eosinophils. (**e**) Conventional dendritic cells were analyzed by flow cytometry for the markers indicated. (**f**) TLR9 expression was examined on CD11b^+^ and CD103^+^ DCs. (**g**, **h**) TLR9 expression was examined on alveolar macrophages (Macs) and lung epithelial cells. (**i**) *Il-12b* and *Hprt* mRNA levels in mouse lung stimulated with CpG oligonucleotide (CpG-ODN) or house dust mite (HDM)*.* (**j**) CD40 and CD86 on lung DCs from indicated mice were analyzed. (**k**) The indicated Mice were sensitized with Alexa647-labeled HDM and analyzed Alexa647-uptaken density on lung and MLN DCs. Data are from three independent experiments (n = 3 to 5 each). (**c–h**) The cells were isolated from lungs in HDM-sensitized mice. Gray histograms in (**b**, **d**, **f**, **g** and **h**) show isotype control staining. Red lines represent NaR9 (anti-mouse-TLR9 monoclonal antibody) staining. WT, wild type. Alx647, Alexa647.

Next, we examined TLR9 expression on lung-infiltrating immune cells, granulocytes, and alveolar epithelial cells in allergic asthma. Compared with the saline administrated group, B cells (B220+) and T cells (CD3^+^) contained in BALF were increased in the HDM administrated group. TLR9 expression was confirmed on B cells, but not on T cells in the BALF from HDM-sensitized mice (Fig. 2a,b). TLR9 was not expressed by either eosinophils or neutrophils (Fig. 2c,d and supplementary Fig. 2b: right panel). Next, cDC subsets were stained for the surface markers CD11c^+^ and MHC class II^+^ (I-A/I-E^+^) (Supplementary Fig. 2b: left panel) and additionally stained with CD11b^+^ or CD103^+^ to separate functionally different cDCs^34,35^. Both CD11b^+^ DCs and CD103^+^ DCs expressed TLR9. Expression on CD11b^+^ DCs was slightly higher than that on CD103^+^ DCs (Fig. 2e,f). Subsequently, surface marker expression on alveolar macrophages (CD11c^+^, CD11b^−^) and lung epithelial cells (CD324^+^/E-cadherin) was examined. Neither alveolar macrophages nor lung epithelial cells showed TLR9 expression (Fig. 2g,h). Finally, we verified the responsiveness of TLR9 to CpG-ODN or HDM. WT and *Tlr9*^*–/–*^ mice were stimulated with CpG-ODN or HDM to analyze *Il12b* expression levels in the lung. IL-12, which is the main cytokine that induces Th1 cell differentiation and activation, is strongly produced in TLR9 response. As expected, in *Tlr9*^*–/–*^ mice we detected no *Il12b* in response to CpG-ODN. In response to HDM, the lung *Il12b* level in *Tlr9*^*–/–*^ mice was significantly lower than that in WT mice (Fig. 2i). Furthermore, in order to evaluate the activation of dendritic cells by HDM, the expression of CD86 or CD40 which are co-stimulators was analyzed. Although CD86 expression was slightly reduced in *Tlr9*^*–/–*^ mice, CD40 expression was almost unchanged from WT mice (Fig. 2j). We also analyzed the uptake of HDM by cDCs. Lungs and MLNs were collected from mice sensitized with Alexa647-labeled HDM, and DCs were analyzed by a flow cytometer. DCs from MLN showed slightly lower Alexa647 fluorescence in *Tlr9*^*–/–*^ mice, but there was not significant difference between WT and *Tlr9*^*–/–*^ DCs in lung and MLN (Fig. 2k). These results suggest that the TLR9 response is involved in the cytokine production resulting from repeated stimulation by HDM.

### Numbers of IL-13- or IL-5-producing Th2 cells are decreased in ***Tlr9***^***–/–***^ mice

**Figure 3:**
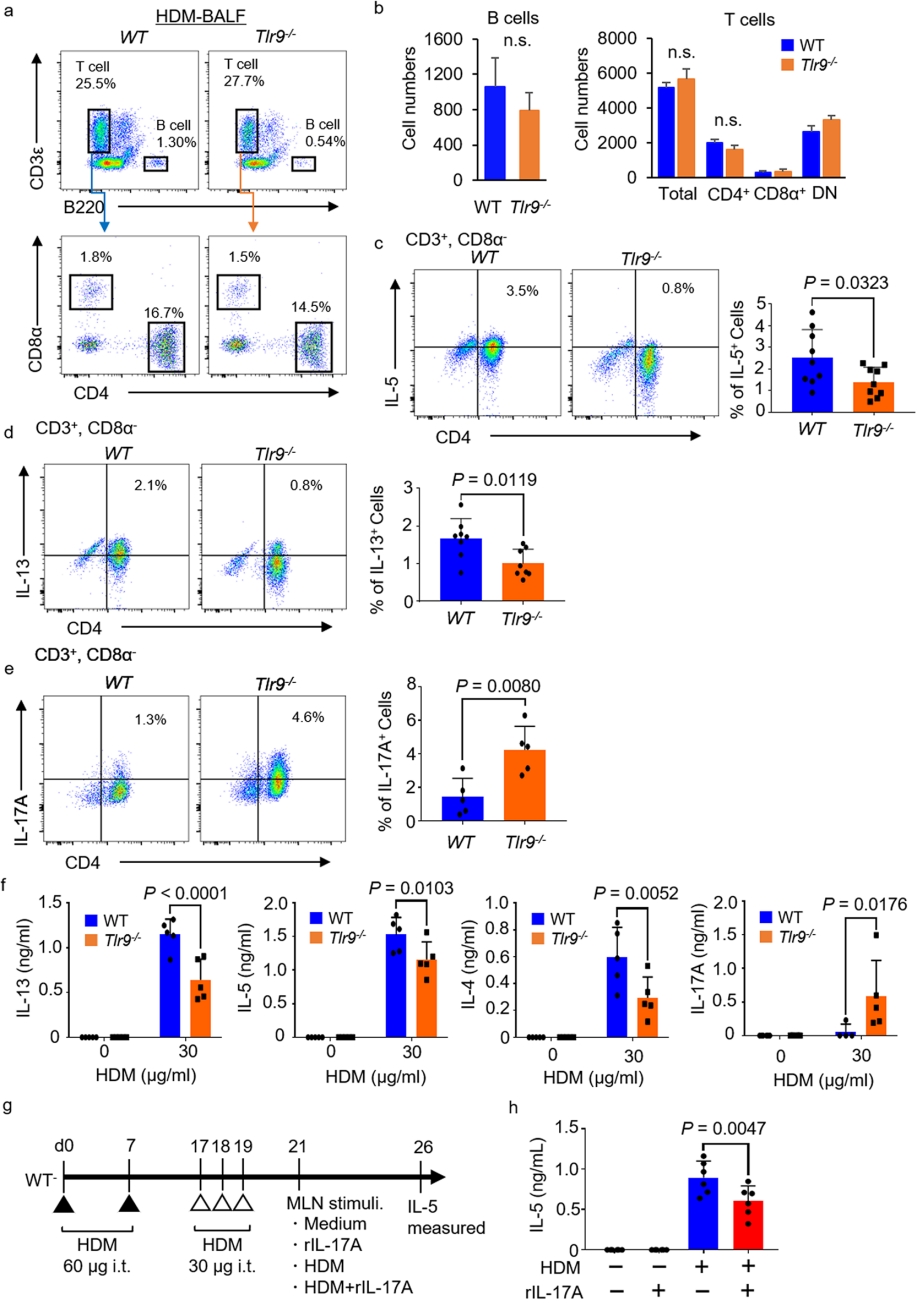
IL-17A production increases in house dust mite (HDM)-treated *Tlr9*^*−/−*^ mice and inhibits IL-5 production. (**a**, **b**) The proportions and numbers B cells and T cells in bronchoalveolar lavage fluid (BALF) were analyzed. (**c**, **d**) CD4^+^ T cells from HDM-sensitized mice were intracellularly stained with anti-IL-5 monoclonal antibody (mAb) or anti-IL-13 mAb and their proportions were analyzed by flow cytometry. (**e**) CD4^+^ T cells from HDM-sensitized mice were intracellularly stained with anti-IL-17A mAb and their proportions were analyzed by flow cytometry. (**f**) Mediastinal lymph node (MLN) cells from HDM-sensitized mice were restimulated with HDM at 10 or 30 μg/mL. Five days later, IL-13, IL-5, IL-4, and IL-17A were quantified in the culture supernatants. (**g**, **h**) MLN cells from HDM-sensitized mice were restimulated with HDM (10 μg/mL) + saline or recombinant IL-17A (rIL-17A). Five days later, IL-5 was quantified in the culture supernatants. Data are presented as means ± SD of two or three independent experiments (n = 3 each). n.s., not significant.

To analyze the pathology of *Tlr9*^*−/−*^ mice, we analyzed BALF cells by flow cytometry. The percentages and numbers of B cells, and of CD4^+^, CD8α^+^, and double-negative T cells, in the BALF were almost the same between WT and *Tlr9*^*−/−*^ mice (Fig. 3a,b). To investigate further, we analyzed the percentage of CD4^+^ Th cells by intracellular staining for IL-5 and IL-13 in CD45^+^, CD3^+^, CD4^+^, Siglec-F^-^ lung cells in HDM-sensitized mice. Similar to the case in the mRNA expression level of IL-5 and IL-13 in allergic asthma (Fig. 1f), the percentages of IL-5^+^ and IL-13^+^ CD4^+^ Th cells were significantly lower in HDM-sensitized *Tlr9*^*−/−*^ mice than in HDM-sensitized WT mice (Fig. 3c,d). In contrast, there was a surprising significant increase in the percentage of IL-17A-producing cells in HDM-sensitized *Tlr9*^*−/−*^ mice (Fig. 3e). We also measured *Il17a* mRNA in lungs sensitized by HDM. To measure IL-17A more precisely, MLN cells were collected from HDM-sensitized mice following restimulation with HDM, and IL-17A in the culture supernatant was quantified. Similar to the results so far, the production amount of IL-13, IL-5 and IL-4 was decreased in MLN cells from *Tlr9*^*−/−*^ mice, but on the other hand, IL-17A production was significantly enhanced in MLN cells from *Tlr9*^*−/−*^ mice (Fig. 3f). Although IL-17A has been reported to be associated with neutrophilic allergic asthma^36^, it has also been reported to suppress the increase in airway resistance caused by intratracheally administered IL-13^37,38^. Therefore, first, to examine the effects of IL-17A, we collected MLN cells from HDM-sensitized WT mice and re-stimulated them with HDM or HDM + IL-17A. The amount of IL-5 was significantly lower in the culture supernatant of MLN cells treated with HDM + IL-17A (Fig. 3g,h). These results suggest that an increase in IL-17A as a result of TLR9 deficiency might suppress Th2 cell activation.

### IL-17A inhibits HDM-induced allergic asthma

**Figure 4:**
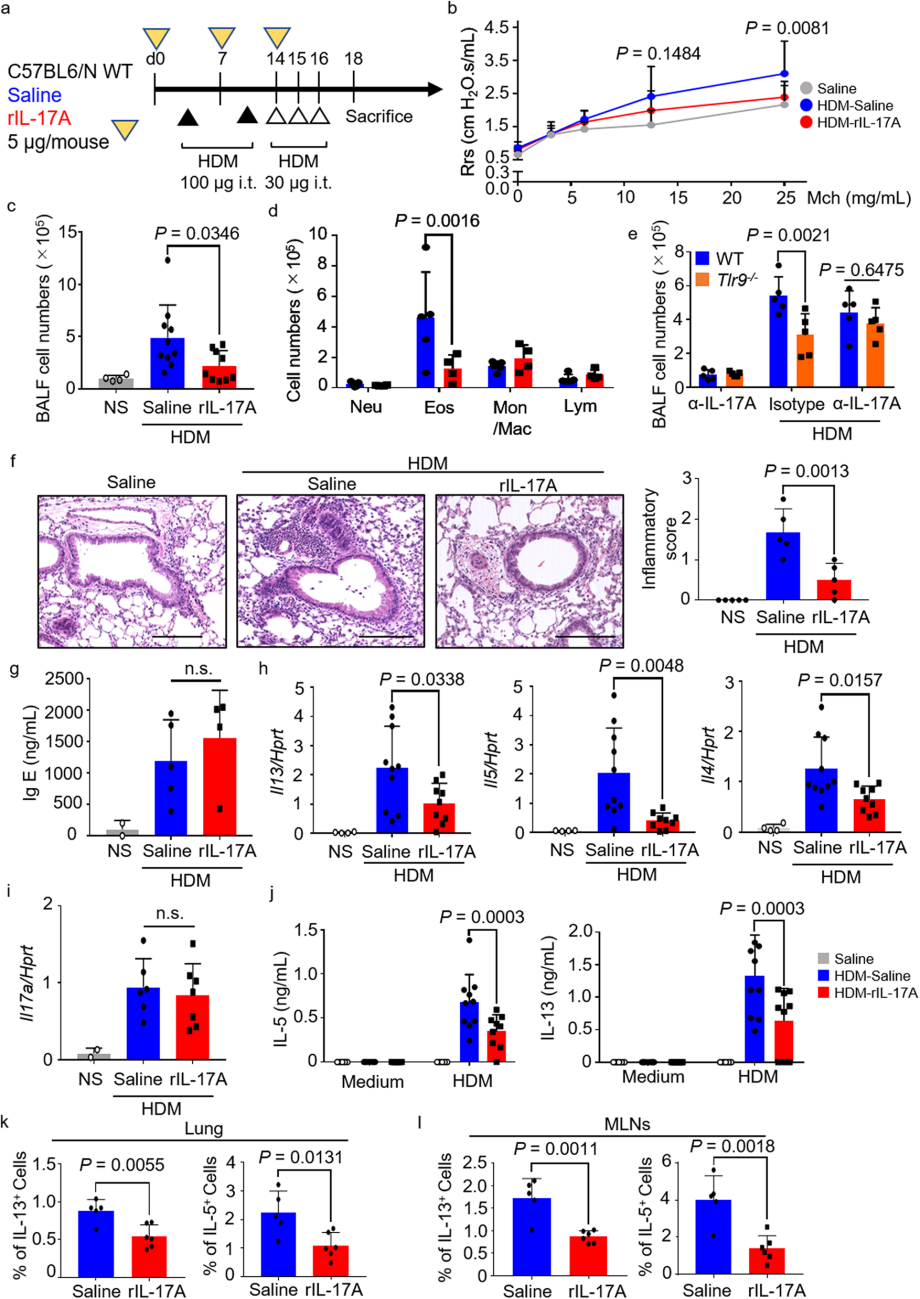
IL-17A suppresses house dust mite (HDM)-induced airway hyperresponsiveness (AHR) and Th2 inflammation. (**a**) Protocol used for treatment with recombinant IL-17A (rIL-17A). (**b**) Airway hyperresponsiveness in the mice indicated was measured by administering methacholine (Mch). Statistical analysis compared between HDM-saline- and HDM-rIL-17A-injected mice. (**c**, **d**) In bronchoalveolar lavage fluid (BALF), total cell numbers and numbers of neutrophils (Neu), eosinophils (Eos), monocytes/macrophages (Mon/Mac), and lymphocytes (Lym) were counted. (**e**) The indicated mice were sensitized with HDM and administrated with anti-mouse IL17A inhibitory antibody or isotype antibody. In bronchoalveolar lavage fluid (BALF), total cell numbers were counted. (**f**) Representative images of hematoxylin and eosin (H&E)-stained histologic sections of mouse lungs. Scale bars, 100 μm. The inflammatory score (0 to 3) was statistically analyzed. (**g**) Serum IgE was measured by ELISA. (**h**) Lung *Il13*, *Il15*, *Il4,* and *Hprt* mRNAs were measured. (**i**) Lung *Il17a* and *Hprt* mRNAs were measured. (**j**) Mediastinal lymph node (MLN) cells from HDM-sensitized mice were restimulated with HDM (10 μg/mL). Five days later, IL-5 and IL-13 in the culture supernatants were measured by ELISA. (**k**, **l**) The percentage of IL-13^+^ and IL-5^+^ cells on CD4^+^ T cells in lung and MLN were statistically analyzed at indicated mice. Data are presented as means ± SD of two or three independent experiments (n = 2 to 5 each). n.s., not significant; i.t., intratracheally; Rrs, respiratory resistance.

Next, we examined whether IL-17A would suppress the pathology of HDM-induced allergic asthma. A previous report had shown that the amount of IL-17A was very important for the inhibitory effect in IL-13-induced AHR^37^. IL-17A at a relatively high dose (5 μg per shot per mouse, total 15 μg/mouse) was intratracheally administered to HDM-sensitized WT mice (Fig. 4a). Administration of IL-17A significantly decreased the increase in AHR in response to HDM, compared with that in the group that received HDM and saline (Fig. 4b). Furthermore, the total number of BALF cells and the number of BALF eosinophils decreased significantly upon administration of IL-17A, but IL-17A did not increase the number of neutrophils (Fig. 4c,d). Next, we used anti-mouse IL-17A neutralizing antibodies to further verify the asthma suppressive effect of the over-producing IL-17A. When HDM-sensitized *Tlr9*^*−/−*^ mice were administered with anti-mouse IL-17A antibody at 40 mg/kg once a week at the same time as the sensitization, the total number of BALF cells from WT mice and *Tlr9*^*−/−*^ mice were almost the same level (Fig. 4e). Pathological analysis confirmed the infiltration of inflammatory cells around the bronchi in the saline + HDM group, whereas almost no cell infiltration was observed in the HDM + IL-17A group (Fig. 4f). However, administration of IL-17A had no effect on IgE production (Fig. 4g). To investigate the allergic status in more detail, we measured the mRNA levels of Th2 cytokines in the lungs. Similar to our other results, *Il13*, *Il5*, and *Il4* mRNA expression was significantly decreased by the administration of IL-17A (Fig. 4h). However, *Il17a* mRNA expression was not altered by the administration of IL-17A (Fig. 4i). Next, MLN cells were collected from HDM-sensitized mice and re-stimulated with HDM. IL-5 and IL-13 in the culture supernatant were measured. IL-17A treatment significantly inhibited IL-5 and IL-13 production by MLN cells (Fig. 4j). Finally, we measured the profile of IL-5^+^ and IL-13^+^ cells from the MLN and lung to clarify t the effect of IL-17A with the aim to clarify the importance of IL-17A for Th2 cell differentiation in the sensitization phase and Th2 cell activation in the challenge phase (Fig. 4k,l). As a result. IL-13^+^ cells and IL-5^+^ cells were decreased in the lung and MLN by the administration of rIL-17A. These results suggest that IL-17A suppresses Th2 cell differentiation of MLN in the sensitization phase and inhibits HDM-induced allergic pathology.

### IL-2 production via the TLR9 response is an important key to the pathogenesis of HDM-induced allergic asthma

**Figure 5:**
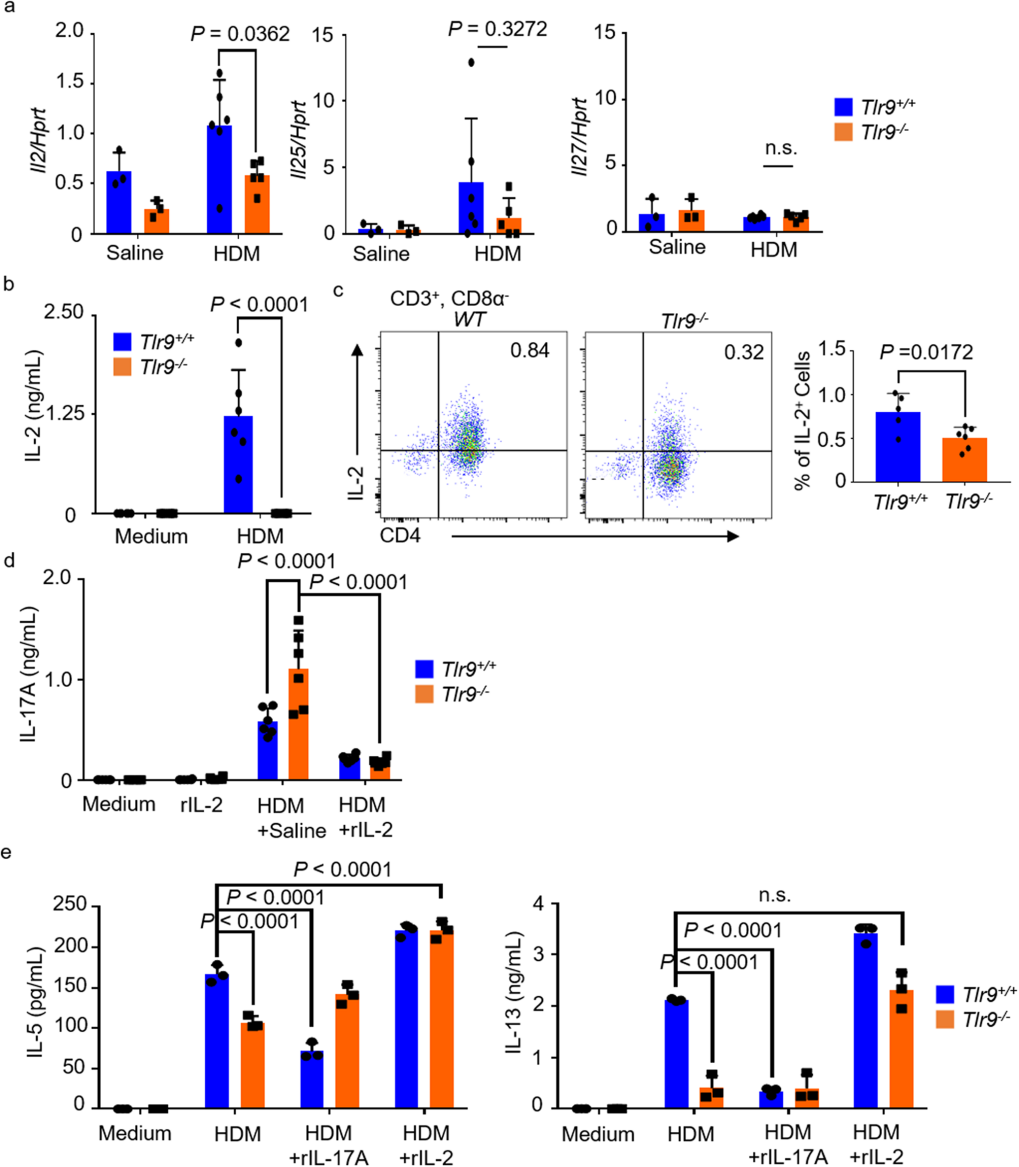
IL-2 production through toll-like receptor 9 (TLR9) response suppresses IL-17A production and promotes Th2 cell activation. (**a**) Lung *Il2, Il25, Il27*, and *Hprt* mRNAs were measured. (**b**) Mediastinal lymph node (MLN) cells from house dust mite (HDM)-sensitized mice were restimulated with HDM (10 μg/mL). Five days later, IL-2 in the culture supernatants was measured by ELISA. (**c**) CD4^+^ T cells from HDM-sensitized mice were intracellularly stained with anti-IL-2 mAb and their proportions were analyzed by flow cytometry. (**d**) MLN cells from HDM-sensitized mice were restimulated with HDM (10 μg/mL) + saline or recombinant IL-2 (rIL-2; 25 ng/mL). Five days later, IL-17A was measured in the culture supernatants by ELISA. (**e**) In the same way as in (**d**), MLN cells from HDM-sensitized mice were restimulated with HDM (10 μg/mL) + saline, or + recombinant IL-17A (rIL-17A; 25 ng/mL), or + rIL-2 (25 ng/mL). Five days later, IL-5 and IL-13 in the culture supernatants were measured by ELISA. Data are presented as means ± SD of three independent experiments (n = 2 or 3 each). n.s., not significant.

Next, we decided to examine how IL-17A production is regulated by TLR9 signaling. IL-1 and IL-23 positively regulate Th17 activation^39,40^, whereas IL-2, IL-25, and IL-27 negatively regulate it^41–43^. We stimulated cDCs (CD11b^+^, CD11c^+^), which were induced from bone marrow cells, by HDM and measured IL-6 and IL-23. IL-6 production differed little between WT mice and *Tlr9*^*−/−*^ mice (data not shown). IL-23 production was not detected (data not shown). Therefore, we conducted an analysis to identify the mechanism of IL-17A hyperproduction, focusing on the factors that negatively regulate Th17 activation. *Il2*, *Il25*, and *Il27* mRNA levels were measured in the lungs of HDM-sensitized mice. *Il2* mRNA expression was significantly lower in HDM-sensitized *Tlr9*^*−/−*^ mice than in HDM-sensitized WT mice. In addition, *Il25* mRNA expression showed a decreasing trend, although there was no significant difference. Almost no *Il27* mRNA was detected in the lungs in either group (Fig. 5a). To investigate IL-2 production in *Tlr9*^*−/−*^ mice, MLN cells collected from HDM-sensitized mice were re-stimulated with HDM, and IL-2 in the culture supernatant was measured. IL-2 was significantly lower in the cells of *Tlr9*^*−/−*^ mice than in those of WT mice (Fig. 5b). To further examine IL-2 producing cells, cytokine staining was performed on Th1 cells, which are a major IL-2 producing subset. As a result, the percentage of IL-2^+^ Th1 cells from *Tlr9*^*−/−*^ mice was significantly lower than that from WT mice (Fig. 5c). Subsequently, we examined whether IL-2 directly affects IL-17A production. IL-17A production was enhanced by HDM re-stimulation of MLN cells from *Tlr9*^*−/−*^ mice. Therefore, at the time of re-stimulation, we added IL-2 and assessed IL-17A production. Addition of IL-2 effectively suppressed IL-17A production by MLN cells from both WT mice and *Tlr9*^*−/−*^ mice (Fig. 5d). Finally, we performed an assay for Th2 cell activation by adding IL-2. When IL-17A was added during re-stimulation with HDM, IL-13 and IL-5 production decreased significantly in MLN cells from WT mice compared with that without IL-17A. In contrast, IL-2 addition to HDM re-stimulation did not reduce IL-13 and IL-5 production in *Tlr9*^*−/−*^ mice (Fig. 5e). These results indicate that IL-2 production from Th1 cells through TLR9 response regulates IL-17A production and thereby activates IL-13- and IL-5-producing Th2 cells.

### NaR9 effectively inhibits immune responses to CpG-ODN and HDM stimulation in the lung

**Figure 6:**
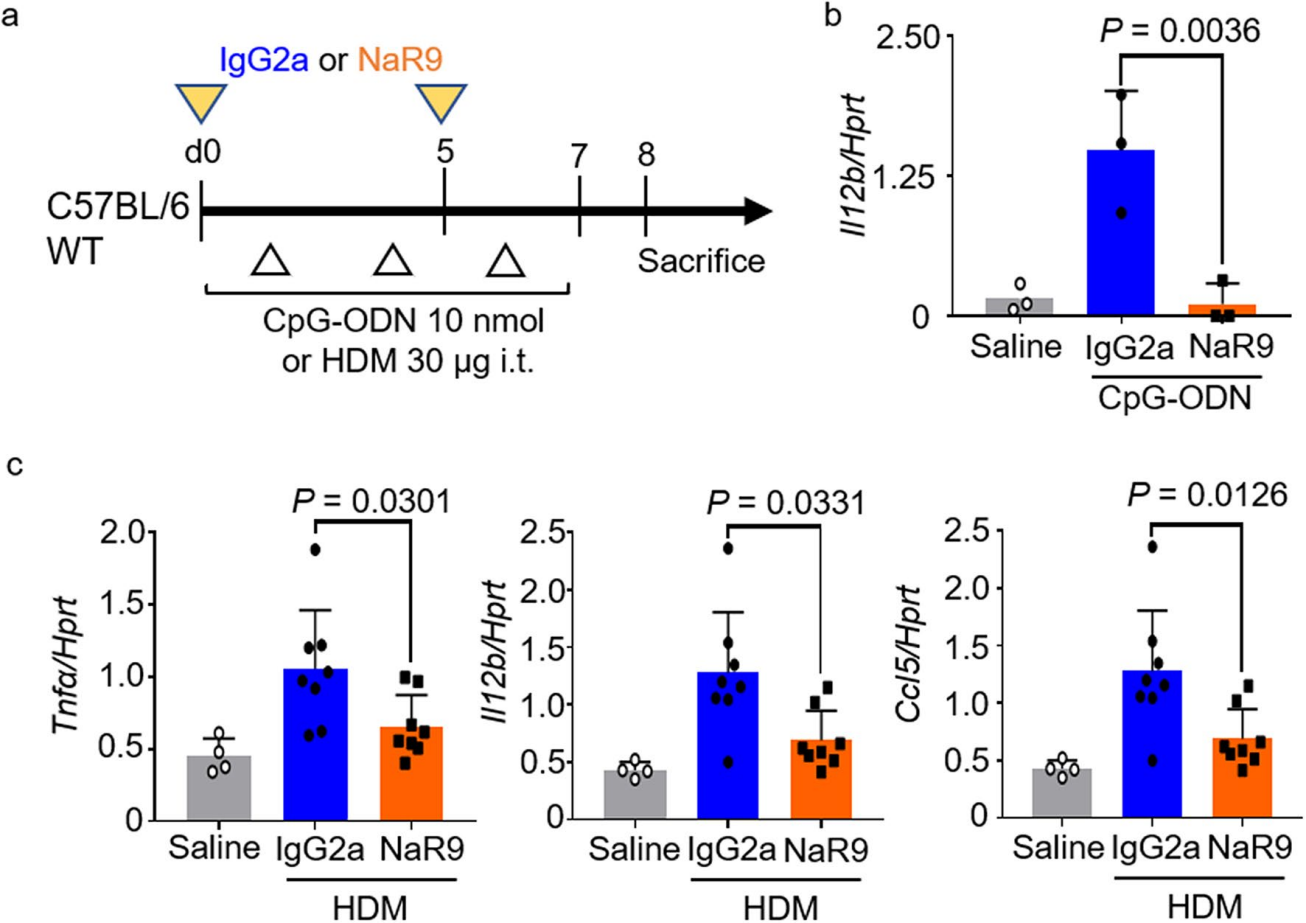
NaR9 inhibits CpG oligonucleotide (CpG-ODN) or short-term house dust mite (HDM) stimulation. (**a**) Protocol used for CpG-ODN or short-term HDM stimulation. (**b**) Lung *Il12b* and *Hprt* mRNAs were measured in mice treated with the anti-mouse-TLR9 monoclonal antibody NaR9. (**c**) Lung *Tnfα*, *Il12b*, *Ccl5,* and *Hprt* mRNAs were measured in mice treated with NaR9 or IgG2a, and sensitized with HDM. Data are presented as means ± SD of two or three independent experiments (n = 2 or 3 each). *n.s.* not significant.

We previously established TLR9 mAb NaR9 that inhibit the TLR9 response in vitro and in vivo liver inflammation^30^. First, we examined whether the TLR9 response in the lung was effectively inhibited by NaR9. CpG-ODN induced *Il12b* mRNA expression in the lungs was significantly lower in NaR9-treated WT mice than in mice treated with IgG2a (Fig. 6b). Furthermore, NaR9 significantly suppressed the increases in expression of *Tnfα*, *Il12b*, and *Ccl5* caused by HDM stimulation (Fig. 6c).

### NaR9 protects from HDM-induced allergic asthma

**Figure 7:**
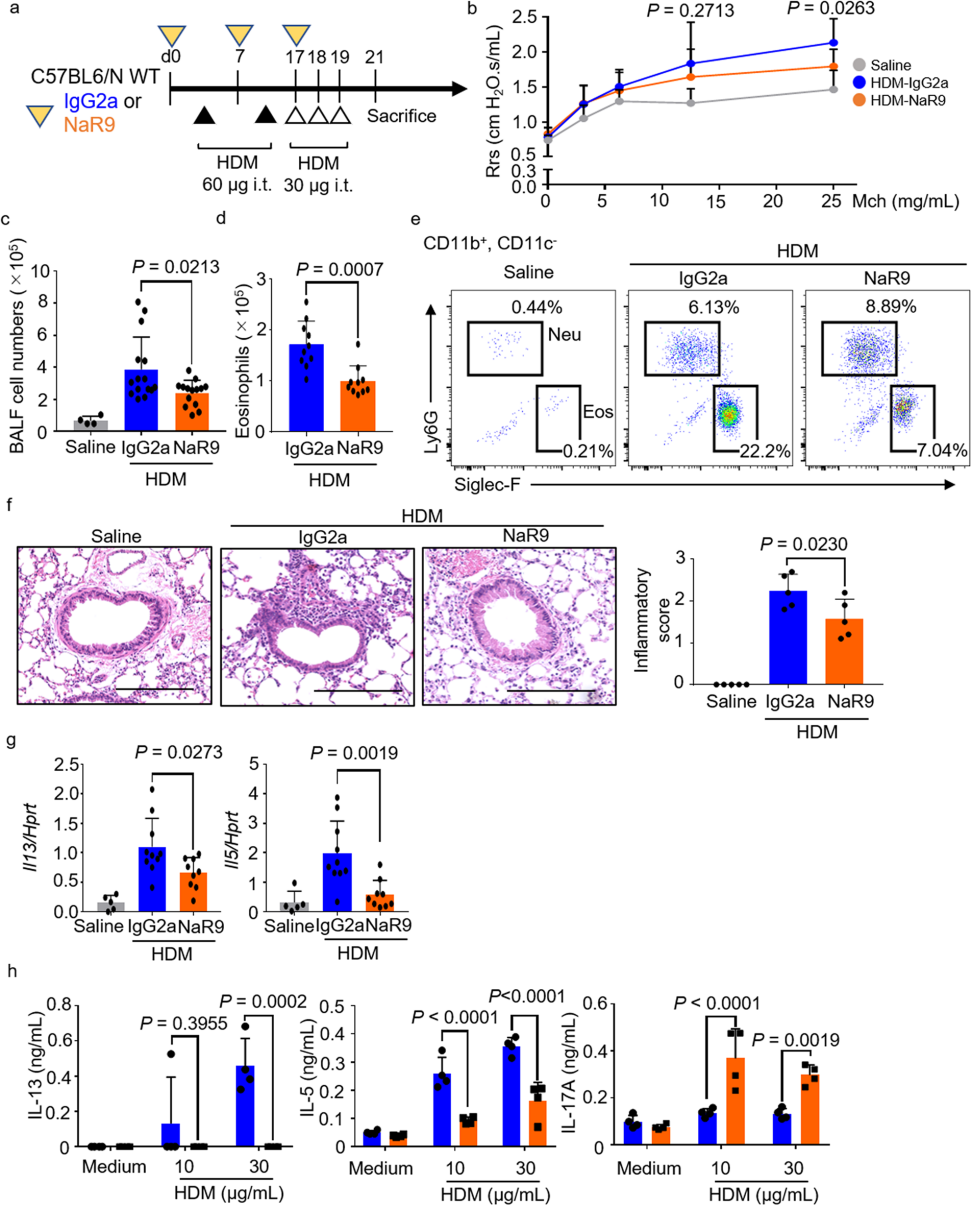
NaR9 effectively protects mice from airway hyperresponsiveness (AHR) and Th2-mediated lung inflammation. (**a**) Protocol used for NaR9 treatment. (**b**) AHR of the mice indicated was measured by administering methacholine (Mch). (**c**, **d**) In bronchoalveolar lavage fluid (BALF), total numbers of cells and numbers of *e*osinophils were counted. (**e**) Proportions of eosinophils and neutrophils in BALF were measured by flow cytometry using the markers indicated. (**f**) Representative images of hematoxylin and eosin stained histologic sections of mouse lungs. Scale bars, 200 μm. The inflammatory score (0 to 3) was statistically analyzed. (**g**) Lung *Il13*, *Il5*, and *Hprt* mRNA levels were measured in the mice indicated. (**h**) Mediastinal lymph node cells from house dust mite (HDM)-sensitized mice were restimulated with HDM (10 or 30 μg/mL). Five days later, IL-5, IL-13, and IL-17a were measured in the culture supernatants by ELISA. Data are presented as means ± SD of three independent experiments (n = 4 or 5 each). *n.s.* not significant, *WT* wild type, *i.t.* intratracheally, *Rrs* respiratory resistance.

Because NaR9 had effect on lung inflammation (Fig. 6), we decided to analyze NaR9 effects on HDM induced asthmatic model (Fig. 7a). Compared with IgG2a, NaR9 significantly suppressed HDM-induced AHR (Fig. 7b). NaR9 also significantly decreased total BALF cell numbers and eosinophil numbers and the percentage of eosinophils in the BALF (Fig. 7c–e). The abundance of immune cells infiltrating around the bronchi and the statistical analysis of the inflammatory score were also effectively decreased (Fig. 7f). NaR9 significantly inhibited *Il13* and *Il5* mRNA expression in the lung (Fig. 7g). Finally, to analyze the activation of Th cells, we collected MLN cells from HDM-sensitized mice and measured cytokine production in response to HDM re-stimulation. IL-13 and IL-5 production significantly decreased upon NaR9 administration, whereas production of IL-17A significantly increased (Fig. 7h). We further analyzed various cytokines, chemokines, and interferons by using the Proteome Profiler (Supplementary Fig. 3). As with our results above, NaR9 suppressed IL-5 production. Additionally, production of RANTES (CCL5) and SDF-1 (CXCL12) was suppressed. In contrast, IL-17A and MIP-2 (CXCL2) production was increased (Supplementary Fig. 3).

### A94B10 does not affect HDM-induced allergic asthma

We decided to determine whether the TLR7 response was involved in the pathology of allergic asthma. We administered normal saline, TLR7 antibody (A94B10) or TLR9 antibody to HDM-sensitized. The number of BALF cells and serum IgE in the TLR7 antibody group was almost the same as that in the control group (Supplementary Fig. 4a,b). These results indicate that the TLR7 response is not involved in the pathology of HDM-induced allergic asthma.

### TLR9 responses are not involved in OVA-alum-induced asthma

Finally, we examined whether the TLR9 response was involved in not only HDM-induced asthma but also OVA-alum-induced asthma. Allergic asthma was induced with OVA-alum (Supplementary Fig. 5a), and the therapeutic effects of anti-TLR7 mAb and anti-TLR9 mAb were determined. There were no differences in the total number of BALF cells or the number of eosinophils infiltrating the lungs (Supplementary Fig. 5b,c). Moreover, there were no differences in the expression levels of *Il13, Il5 and Il17a* mRNA in the lungs (Supplementary Fig. 5d). These results suggest that the effect of the TLR9–IL-2 axis is specific for HDM-induced allergic asthma.

## Discussion

Here, we showed that TLR9 promotes Th2-induced AHR and lung inflammation by preventing IL-17A hyperproduction via IL-2 suppressive effects. Importantly, TLR9 not only affects Th1 activation, but also indirectly controls Th2 and Th17 activation. The TLR9–IL-2 axis performs a critical effector role to produce the pathology of HDM-induced allergic asthma. We further showed here that inhibition of the TLR9 response by our originally established anti-mouse TLR9 monoclonal antibody, also suppressed HDM-induced AHR and type 2 inflammation. Notably, the ability to induce IL-17A hyperproduction, not only in *Tlr9*^*−/−*^ mice but also upon treatment with NaR9, indicates that monoclonal antibody administration is effective as a means of controlling the activation and differentiation balance of Th cells.

It is well known that the pathological condition of allergic asthma develops through infiltration of eosinophils, type II cytokines produced by Th2 or ILC2, and inflammatory mediators released from mast cells and basophils. Also, in airway epithelial cells, alarmins such as IL-33, IL-25, and thymic stromal lymphopoietin (TSLP) work as not only an initial reaction of allergic pathology but ILC2 activators^1,44,45^. Given that nucleic-acid-sensing TLRs are expressed mainly in immune cells and activate Th1 cells, it may be difficult for the TLRs to directly promote Th2 allergic conditions. For this reason, we focused here on an HDM-dependent and TLR9-driven mechanism for suppressing the airway and lung inflammation triggered by Th2 cells. This differs from previous studies that have investigated the suppressive role of TLR9 agonists^18,19^.

In our study, *Tlr9*^*−/−*^ mice showed significant suppression of allergic inflammation induced by HDM. This suggests that TLR responses to HDM-derived ligands and self-nucleic-acid ligands exacerbate the pathology of allergic asthma. It also further emphasizes the importance of treatments targeting TLRs in asthma.

Indeed, HDM contains TLR4 ligands such as LPS, and the cytokine production from BM-DCs is largely TLR4 dependent (Supplementary Fig. 1a). On the other hand, the cytokine production by DNase-treated HDM extract is lower than that by DNase-untreated HDM extract in BM-DCs from *Tlr4*^*−/−*^, so it is concluded that HDM-derived DNA can partially stimulated DNA receptors.

The mechanism of the asthma-suppressive effect in *Tlr9*^*−/−*^ mice relied on the over-induction of IL-17A, creating a situation of Th17 activation that antagonized Th2 inflammation. Furthermore, the relationship between IL-17 and asthma has been reported in TLR other than TLR9. It has been reported that *Tlr6*^*−/−*^ mice show exacerbation of fungal-induced allergic asthma because of low levels of IL-23 and IL-17A^46^. IL-17A has been reported to suppress Th2 activation and IL-13-induced airway hyperresponsiveness^37,38^. A report of IL-13-induced AHR states that the concentration of IL-17A is very important for its inhibitory effect: single doses of 0.5 μg/mouse, totaling 4 μg IL-17A, do not affect AHR, but 1.5 μg/mouse (totaling 12 μg IL-17A) can effectively decrease AHR^37^. In our study, a total of 15 μg IL-17A significantly reduced HDM-induced AHR. Although IL-17A does not directly affect T-cell activation, it has been reported to inhibit allergic inflammation by suppressing DC activation^38^. On the other hand, there are conflicting reports in this regard and this remains a topic of discussion^36,40^.

Focusing on TLR9 expression, B cells as well as dendritic cells may influence T cell activation. Since it has been reported that the activation of antigen-specific T cells depends on B cells^47^, it is necessary to analyze B cell antigen presentation and activation in *Tlr9*^*−/−*^. In addition, there have been many reports of γδ T cells as IL-17A-producing cells^48^, and IL-17A produced by γδ T cells suppresses AHR and eosinophilic inflammation^49^. Because TLR9 is not expressed on γδ T cells, it is currently unknown whether there is a pathway that regulates γδ T-cell activation downstream of TLR9 signaling. In future, we need to analyze the role of γδ T cells for HDM-induced allergic asthma in *Tlr9*^*−/−*^ mice.

To elucidate the mechanism of IL-17A hyperproduction, IL-17A production regulators have been investigated. Positive regulators include TGF-β, IL-1β, IL-6, and IL-23^39,40^. Negative regulators include IL-2, IL-25, and IL-27^42,43^. Considering the hyperproduction of IL-17A in *Tlr9*^*−/−*^ mice, the cause may be a decrease in negative regulators. Indeed, MLN cells from HDM-sensitized mice treated with NaR9 did not differ in IL-1β, IL-6, or IL-23 production compared with the control group (Supplementary Fig. 3). However, the mRNA expression of IL-2 was reduced in HDM-sensitized *Tlr9*^*−/−*^ mice compared with WT mice. Considering that IL-2-producing cells are Th1 cells, it is likely that Th1 cells induce IL-2 production downstream of TLR9 signaling. The TLR–IL-12 axis induces Th1 differentiation and activation^50^. Although IL-2 is a Th1 cytokine, it is also important in the development of allergic diseases^51,52^.

Although Th1 also produces IFN-γ through the TLR–IL-12 axis, *Ifng* mRNA expression levels in inflamed lungs were not different between WT and *Tlr9*^*−/−*^ mice. There is a possibility that NK cells, which secrete a high level of IFN-γ, may produce IFN-γ instead of Th1 cells in our allergic asthma model^53,54^.

To evaluate the therapeutic effects of TLR9 inhibitory antibody, NaR9 in allergic asthma, we gave NaR9 to allergic asthma model mice. NaR9 had until now been confirmed only to inhibit the TLR9 response in the liver^30^, so this is the first verification of the inhibitory effect in the lung. NaR9 almost completely inhibited the *Il12b* mRNA expression caused by CpG-ODN stimulation (Fig. 6b), suggesting that it has a suppressive effect of TLR9 response in the lung.

HDM induces complex immune responses because it contains LPS and other immunostimulatory ligands. In our study using bone-marrow-derived DCs, at low concentrations of HDM (10 μg/mL) CCL5 production by *Tlr4*^*−/−*^ cells disappeared, but in *Tlr9*^*−/−*^ cells it decreased by about half compared with that in WT cells. Furthermore, stimulation with a high concentration of HDM (30 μg/mL) had no inhibitory effect on *Tlr9*^*−/−*^ cells (Supplementary Fig. 1a). This result indicates that the TLR9 response to HDM-derived DNA is partial in the immune response of DCs. Surprisingly, CCL5 production was increased by HDM in the cytoplasmic DNA sensor *Sting*^*−/−*^ cells (Supplementary Fig. 1B). STING has been reported not only to function as a DNA sensor but also to negatively regulate TLR7 and TLR9 responses in lupus-prone mice^55^. Given these results, *Sting*^*−/−*^ cells may have an enhanced response of TLR9, TLR4, or other innate immune receptors to HDM. In contrast to DCs, NaR9 effectively suppressed the expression of *Ccl5*, *Tnfα*, and *Il12b* mRNAs of lung from HDM-treated mice (Fig. 6c). Reflecting this result, NaR9 effectively suppressed allergic inflammation and also induced IL-17A hyperproduction. These results suggest that the TLR9 response to HDM-derived DNA is only partial and that the response to self-DNA is largely involved in the pathology.

Our final conclusion is that TLR9 may be an effective therapeutic target in HDM-induced allergic asthma: treatment with anti-TLR9 inhibitory antibody abruptly increased IL-17A production by interfering with the TLR9–IL-2 axis. Notably, it is very interesting that the pathology of allergic asthma is based on an exquisite balance of Th1, Th2, and Th17 cell activation. Our results indicate that the blocking monoclonal antibody for the human TLR9 response will be promising therapeutic methods for controlling the balance of Th1, Th2 and Th17 to improve asthmatic patients.

## Methods

### Study approval

All experiments of this study were approved by the Animal Research Committee (approval numbers 05-A-2017, 05-A-2018, 05-A-2019) and the DNA Biosafety Committee (approval numbers ExH29-#5, ExH30-#5, ExH31-#5, ExR01-#5) of Musashino University and performed in accordance with Japan’s and the University’s guidelines.

### Mice

C57BL/6N mice were purchased from Sankyo Labo Service Corporation, Inc. (Tokyo, Japan). *Tlr4*^*−/−*^ mice were purchased from Oriental Bio Service (Kyoto, Japan). *Tlr9*^*−/−*^ mice on a C57BL/6 background were kindly provided by Dr. Kensuke Miyake’s laboratory (University of Tokyo, Institute of medical science, Minato-ku, Tokyo, Japan). *Sting*^*−/−*^ mice on a C57BL/6 background were kindly provided by Dr. Glen N. Barber’s laboratory (University of Miami, Miller School of Medicine, Miami, FL, USA).

### Allergic asthma induction

HDM extract was kindly produced by the Institute of Tokyo Environmental Allergy, Inc. (Tokyo, Japan). The method for HDM-induced allergic asthma was optimized from our previous reports^9,56,57^.

and another article^7^. C57BL/6N mice were intratracheally administered HDM extract at 100 μg or 60 μg/head on days 0 and 7 and then at 30 μg/head on days 17, 18, and 19 (see Fig. 1A). They were sacrificed on day 21 while under anesthesia with ketamine (90 mg/kg) (Daiichi-Sankyo Co. Ltd. Shinagawa, Japan) and medetomidine (1 mg/kg) administered via intraperitoneal injection. Mice treated with normal saline (Ootsuka; Tokushima, Japan) were used as controls.

The method for OVA-Alum-induced allergic asthma was used from our previous report^56^. C57BL/6N mice were sensitized intraperitoneally with 2 μg of ovalbumin from Sigma-Aldrich (OVA; A5503, Saint Louis, MO, USA) in conjunction with aluminum adjuvant in 0.5 mL of phosphate-buffered saline on days 0 and 14. One week after the second sensitization, the mice were challenged with either aerosolized saline or OVA (20 mg/mL saline) intranasally for 10 min on each of 5 days in a closed chamber by using a MicroMist nebulizer (DeVilbiss, PA, USA).

### Alexa647-labeled HDM

HDM extract was labeled Alexa647 by Alexa Fluor™ 647 Protein Labeling Kit (Thermo Fisher Scientific, Minato-ku, Tokyo, Japan).

### Measurement of airway hyperresponsiveness (AHR)

AHR was examined 48 h after the final administration of HDM extract by using a flexiVent system (Scireq, Montreal, Quebec, Canada)^56^. The mice were tracheostomized while under anesthesia with ketamine (90 mg/kg) and medetomidine (1 mg/kg) administered via intraperitoneal injection. The animals were ventilated at a tidal volume of 8 mL/kg and respiratory frequency of 150/min with a positive end expiratory pressure of 2 cm H_2_O. After the mice had been placed on ventilation, they were injected with rocuronium bromide. To assess AHR, methacholine (3.12, 6.25, 12.5, or 25 mg/mL) was administered by means of nebulization. Airway resistance as a measure of AHR was determined by using the Snapshot-150 perturbation maneuver method of the flexiVent system.

### Analysis of bronchoalveolar lavage fluid (BALF)

Forty-eight hours after the final administration of HDM extract, whole blood was collected via heart puncture or cheek veins into a heparinized syringe, and BALF cell analysis was performed as reported previously^9,57,59^. BALF was obtained by washing the lungs twice with 1 mL of sterile normal saline and was centrifuged at 540×*g* for 5 min at 4 °C. The pellets were resuspended in 1 mL of normal saline and the number of cells counted. Cytospin preparations were stained with Diff-Quik (International Reagents Corporation, Osaka, Japan) and then examined under an optical microscope.

### Histological analysis and evaluation

Lungs were removed from HDM-sensitized mice and infiltrated with 10% formalin neutral buffer solution for fixation. The lungs were then embedded in paraffin wax for slicing. The slices were stained with hematoxylin and eosin (HE) or periodic acid–Schiff (PAS) and analyzed under an EVOS microscope (Thermo Fisher Scientific, Minato-ku, Tokyo, Japan) or under a BZ-8100 fluorescence microscope (Keyence, Osaka, Japan). The extent of perivascular and peribronchial inflammation was estimated by a score calculated by means of quantification of inflammatory cells in lung sections stained with HE. Briefly, score 0 was assigned to bronchi with no surrounding leukocyte infiltration; score 1 corresponded to few infiltrating leukocytes; scores 2 or 3 were assigned if there were from 1 to 2 or 3 to 5 layer(s) of perivascular and/or peribronchial leukocytes, respectively. Mucus production was quantified as the percentage of PAS–stained goblet cells per total epithelial cells in randomly selected bronchi.

### RNA extraction and complementary DNA (cDNA) synthesis

The method for RNA extraction was demonstrated from our previous reports^9,58^. Lungs from HDM- or normal-saline-treated mice were incubated with Sepasol-RNA I Super G solution for RNA isolation (Nacalai Tesque, Osaka, Japan) and then homogenized with metal beads in a multi-bead shocker (Yasui Kikai, Osaka, Japan). The crushed samples were added to 200μL chloroform and then centrifuged at 15,300×*g* at 4 °C for 15 min. The supernatant was collected and 500 μL isopropanol added. The sample was then mixed well, and the mixture was centrifuged at 15,300×*g* at 4 °C for 10 min. The supernatant was removed and 70% ethanol added to the nucleic acid pellet. The pellet was then centrifuged at 15,300×*g* at 4 °C for 5 min. The supernatant was removed and the nucleic acid pellet dried and dissolved in 200 μL water.

The method for cDNA synthesis was demonstrated from our previous reports^28,30^. cDNA was synthesized from the extracted RNA by ReverTra Ace qPCR Master Mix (Toyobo, Osaka, Japan).

### Real-time polymerase chain reaction

The method for Real-time PCR was referenced from our previous reports^28,30,60^. For measurement of mRNA levels, cDNA was quantified by means of real-time polymerase chain reaction using TaqMan probes and primers (indicated below) (Sigma Aldrich Japan, Meguro, Japan) in a total volume of 20 μL. *Tnfα* and the Il12b probe and primer pair mixes were purchased from Roche (Basel, Switzerland). Expression of genes of interest was calculated according to the comparative threshold cycle method, using the hypoxanthine–guanine phosphoribosyltransferase gene (*Hprt*) as an internal control.

The following forward and reverse primers and probe were used:

*Il13* probe, 5′-CCGGTGCCAAGATCTGTGTCTCTCCCT-3′

*Il13* forward, 5′-TGGCTCTTGCTTGCCTTGG-3′

*Il13* reverse, 5′-GTTGCACAGGGGAGTCTGG-3′

*Il5* probe, 5′-AAAGAGAAGTGTGGCGAGGAGAGACGGAG-3′

*Il5* forward, 5′-GGGGTACTGTGGAAATGCTATTC-3′

*Il5* reverse, 5′-CTTGCAGGTAATCCAGGAACTG-3′

*Il4* probe, 5′-TCCTCACAGCAACGAAGAACACCACAGA-3′

*Il4* forward, 5′-CACGGAGATGGATGTGCCAAA-3′

*Il4* reverse, 5′-GCGAAGCACCTTGGAAGCC-3′

*Ifng* probe, 5′-GGAGGAACTGGCAAAAGGATGGTGACATGA-3′

*Ifng* forward, 5′-GGCATAGATGTGGAAGAAAAGAGTC-3′

*Ifng* reverse, 5′-GAGGTAGAAAGAGATAATCTGGCTC-3′

*Il17a* probe, 5′-CGGCTACAGTGAAGGCAGCAGCGAT-3′

*Il17a* forward, 5′-GGAGAGCTTCATCTGTGTCTCTG-3′

*Il17a* reverse, 5′-GGACACGCTGAGCTTTGAGG-3′

*Il2* probe, 5′-CTGAAACTCCCCAGGATGCTCACCTTCAAA-3′

*Il2* forward, 5′-AGCAGGATGGAGAATTACAGGA-3′

*Il2* reverse, 5′-CCAGAACATGCCGCAGAGG-3′

*Ccl5* probe, 5′-GACCACTCCCTGCTGCTTTGCCTACCT-3′

*Ccl5* forward, 5′-CGCACCTGCCTCACCATATG-3′

*Ccl5* reverse, 5′-CGGTTCCTTCGAGTGACAAACA-3′

*Il25* probe, 5′-CAGGACCTGTACCACGCTCGATGCCT-3′

*Il25* forward, 5′-ATCTCTCCTTGGAGCTATGAGTTG-3′

*Il25* reverse, 5′-AAGTGGGACGGAGTTGCCC-3′

*Il27* probe, 5′-AGGCATGGCATCACCTCTCTGACTCTGAG-3′

*Il27* forward, 5′-CTTCCCAATGTTTCCCTGACTTTC-3′

*Il27* reverse, 5′-GTGTGGTAGCGAGGAAGCAG-3′

*Hprt* probe, 5′-AGCTTGCTGGTGAAAAGGACCTCTCGAAGT-3′

*Hprt* forward, 5′-CAGCCCCAAAATGGTTAAGGTTG-3′

*Hprt* reverse, 5′-CCAACAAAGTCTGGCCTGTATCC-3′.

### Reagents and antibodies

The reagents and antibodies used in this study were referenced from our previous reports^28–30,32,60^. Lipopolysaccharide (LPS) from *Escherichia coli* (LPS O55:B5, L2880) was purchased from Sigma-Aldrich (MO, USA). Pam3csk4, CpG-ODNs and cGAMP (cyclic guanosine monophosphate–adenosine monophosphate) were purchased from Invivogen (CA, USA). Murine GM-CSF (granulocyte–macrophage colony-stimulating factor) was purchased from Peprotech (Rocky Hill, NJ, USA). The anti-mouse TLR7 mAb A94B10 and the anti-TLR9 mAb NaR9 were purified from ascitic fluid, as reported previously^29,30^. Streptavidin–phycoerythrin (PE), anti-mouse IgG1-PE, anti-mouse IgG2a-PE, isotype control antibodies (mouse IgG1, mouse IgG2a), and antibodies against mouse CD16/32, CD3-PE-Cy7, CD45-APC-Cy7, B220-FITC, CD11b-APC, CD11c-BV421, CD11c-PE-Cy7, Siglec-F-FITC, Ly-6G-FITC, CD4-BV510, CD8α-Percp/Cy5.5, CCR3-Percp/Cy5.5, I-A/I-E-BV510, CD103-FITC and IL17A-APC were purchased from BioLegend (San Diego, CA, USA). Anti-mouse IL5-PE, anti-mouse IL-2-APC, and anti-mouse IL13-eFluor450 were purchased from Invitrogen (Thermo Fisher Scientific, Minato-ku, Tokyo, Japan). Mouse IL-17A and IL-2 recombinant proteins were purchased from BioLegend.

### Recombinant mouse IL-17A treatment

Murine recombinant IL-17A (rIL-17A) was administered intratracheally at 5 μg per mouse once a week for a total of three times (days, 0, 7, and 14) (Fig. 4A). rIL-17A was mixed to HDM extract and injected intratracheally at the same time.

### Anti-mouse IL-17A inhibitory antibody treatment

In HDM-induced allergic asthma, C57BL6N mice and *Tlr9*^*−/−*^ mice were injected intraperitoneally with 40 mg/kg of anti-mouse IL-17A antibody (BioXCell, Lebanon, NH, USA) or control IgG1 monoclonal antibodies three times in 2 weeks (days 0, 7, 17) to block IL-17A. A total of 10–12 mice were used in each group.

### Anti-mouse TLR9 inhibitory antibody treatment

In the short-term stimulation assay, mice were injected intraperitoneally with 40 mg/kg of anti-mouse TLR9 (NaR9) or control IgG2a monoclonal antibody twice in 1 week (days 0 and 5) to block TLR9 responses. In HDM-induced allergic asthma, C57BL6N mice were injected intraperitoneally with 40 mg/kg of anti-mouse TLR9 (NaR9) or control IgG2a monoclonal antibodies three times in 2 weeks (days 0, 7, 17) to block TLR9 responses. A total of 10–15 mice were used in each group.

### Cell culture

Mediastinal lymph node (MLN) cells were cultured in Roswell Park Memorial Institute 1640 medium (Nacalai Tesque) supplemented with 10% fetal bovine serum (BioWest, Kansas City, KS, USA), antibiotic–antimycotic mixed stock solution (100 ×) from Nacalai Tesque, and 50 μM 2-Mercaptoethanol (ME) from Wako (Tokyo, Japan). MLN cells from HDM-sensitized mice were re-stimulated with HDM at 10 or 30 μg/mL. Five days later, IL-13, IL-5, IL-4, IL-2 and IL-17A in the culture supernatants were quantified by ELISA. Mouse conventional DCs (cDC) were induced from Bone-marrow cells of WT, *Tlr4*^*−/−*^, *Tlr9*^*−/−*^, *Sting*^*−/−*^ mice by the incubation with 100 ng/mL GM-CSF for 7 days.

### ELISA

IL-2, IL-4, IL-5, IL-12 p40, IL-13, and IL-17A were detected in the culture supernatants of HDM-stimulated mediastinal lymph node (MLN) cells from HDM-sensitized mice by using an ELISA MAX Deluxe Set (BioLegend). IgE was detected in the sera of HDM-sensitized mice by using an ELISA MAX Deluxe Set (BioLegend).

### Proteome profiler

Multiple cytokines were detected in the culture supernatants of HDM-stimulated MLN cells from HDM-sensitized mice by using a Proteome Profiler (R&D, Minneapolis, MN, USA).

### Cell staining and flow cytometry

The methods for cell staining and flow cytometry were referenced from our previous reports^28,30,60^. For staining of infiltrating cells in BALF, a single-cell suspension was prepared, and red blood cells were lysed in RBC lysis buffer (BioLegend). The Fc receptor on prepared cells was blocked by anti-CD16/32 (BioLegend). The cells were then subjected to counterstaining below. Staining for B220, CD3, CD4, and CD8a was conducted to separate B cells and T-cell subsets. Eosinophils and neutrophils in BALFs were divided by staining with mAbs for the surface markers such as CD11b, Siglec-F, CCR3, CD11c, and Ly6G and then permeabilized to confirm TLR9 expression. Staining for NK1.1, CD11b, CD11c, Ly6C, and Ly6G was conducted to separate monocyte subsets. Staining for CD11c, I-A/I-E, CD11b, and CD103 was conducted to separate DC subsets. Staining for CD3, CD4, CD8a, and Siglec-F was conducted to separate helper T cell subsets. After being counterstained, the cells were fixed with Fixation/Permeabilization buffer (BD Biosciences, Franklin Lakes, NJ, USA) for 20 min at 4 °C. The cells were then washed twice with 1 × Perm/Wash buffer (BD Biosciences) and incubated in 200 ng/mL of anti-IgG2a or NaR9, or in 2 μg/mL of anti-IL-2, anti-IL-5, anti-IL-13 or anti-IL-17A for 30 min at 4 °C. Finally, the cells were washed twice with 1 × Perm/Wash buffer and suspended in a staining buffer (10% FCS, 0.1% Sodium azide in 1 × PBS) for flow cytometry. Prepared cells were subjected to flow cytometry analysis by FACSLyric (BD Biosciences). Flow cytometry data were analyzed by using FlowJo software (Ashland, OR, USA) (Supplementary Fig. 2A).

### Statistical analysis

Statistical significance was calculated by performing a two-tailed unpaired Student’s *t*-test or one-way ANOVA. A *P-*value of < 0.05 was considered statistically significant.

## Supplementary information

Supplementary Information.
