# Supplementary material for: TLR9–IL-2 axis exacerbates allergic asthma by preventing IL-17A hyperproduction
Source: Sci Rep. 2020 Oct 22;10:18110. doi: 10.1038/s41598-020-75153-y (PMC7581806; doi:10.1038/s41598-020-75153-y)
Supplement: Supplementary file 1 — Supplementary Information. [file 41598_2020_75153_MOESM1_ESM.pdf]

Title: mTLR9–IL-2 axis exacerbates allergic asthma by preventing IL-17A hyperproduction

Authors: Yusuke Murakami[1], Takashi Ishii[1], Hiroki Nunokawa[1], Keigo Kurata[2], Tomoya Narita[1], and Naomi Yamashita[1]

Affiliations:

1, Faculty of Pharmacy, Department of Pharmaceutical Sciences, Musashino University, Nishitokyo-shi, Tokyo, Japan

2, Institute of Tokyo Environmental Allergy, Inc., Tokyo, Japan

Corresponding author: Naomi Yamashita, MD, PhD,

Faculty of Pharmacy, Musashino University, Nishitokyo-shi, Tokyo, 202-8585, Japan.

Tel: +81-42-468-8647

Email: naoyama@musashino-u.ac.jp

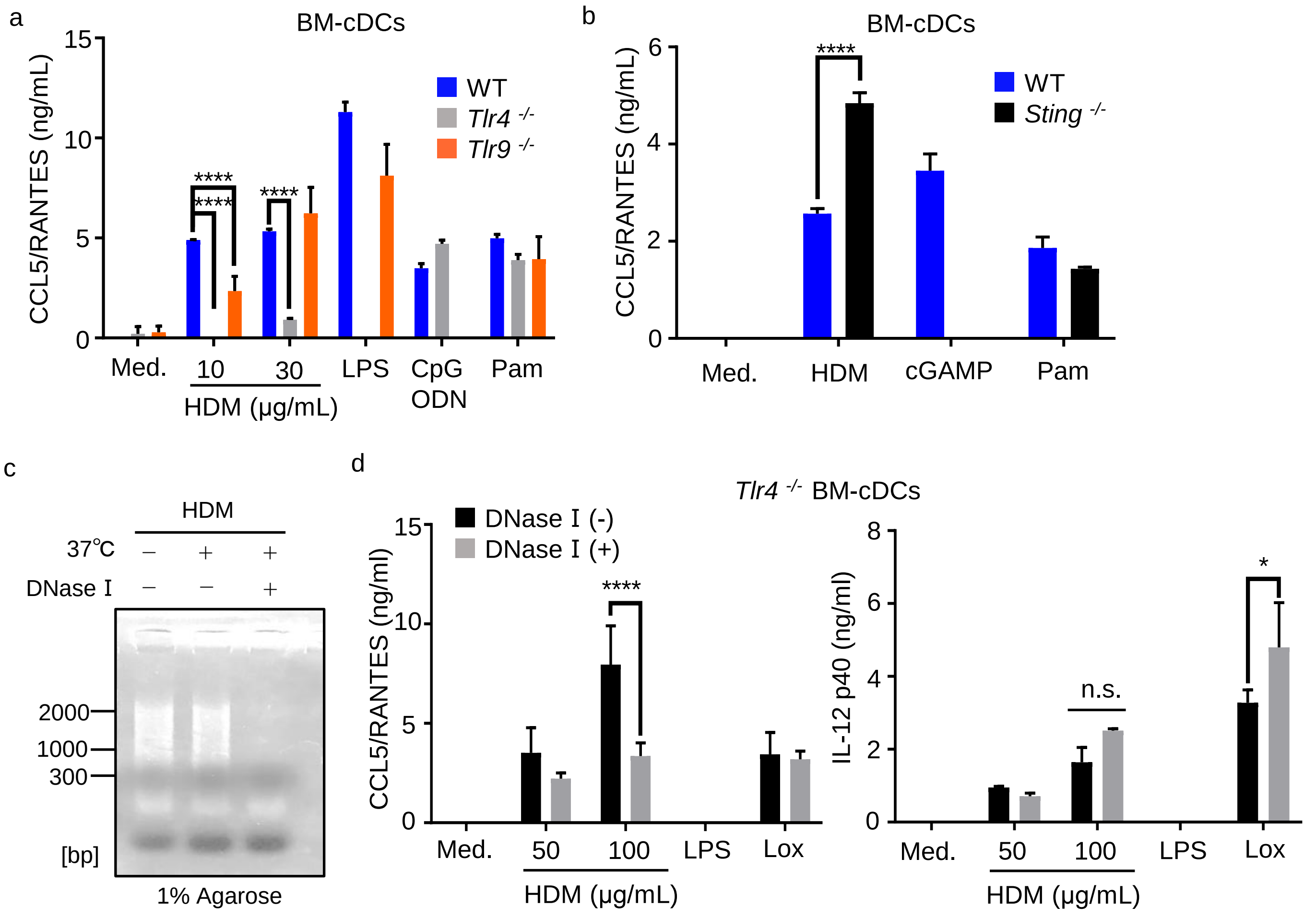

Figure S1. House dust mite (HDM) DNA partially stimulates Toll-like receptor 9 (TLR9) in bone-marrow-derived dendritic cells. (a) Bone-marrow-derived conventional dendritic cells (BM-cDCs) from wild-type (WT), *Tlr4* $^{-/-}$, or *Tlr9* $^{-/-}$ mice were stimulated with HDM (10 or 30 μg/mL), lipopolysaccharide (LPS; 10 ng/mL, CpG oligonucleotide (CpG-ODN; 100 nM), or Pam3CSK4 (Pam; 100 ng/mL). Twenty-four hours later, the CCL5/RANTES concentration in the culture supernatants was measured by ELISA. (b) BM-cDCs from wild-type (WT) or *STING* $^{-/-}$ mice were stimulated with HDM (10 μg/mL), cGAMP (25 μg/mL), or Pam3CSK4 (100 ng/mL). Twenty-four hours later, the CCL5/RANTES concentration in the culture supernatants was measured by ELISA. (c) HDM was left untreated or treated with DNase I and incubated at 37 °C. The samples were then electrophoresed in 1% agarose gel and double-stranded DNA bands detected. (d) BM-cDCs from *Tlr4* $^{-/-}$ mice were stimulated with DNase I-treated or untreated HDM (50 or 100 μg/mL), LPS (10 ng/mL), or loxoribine (Lox; 250 μM). Twenty-four hours later, the CCL5/RANTES and IL-12 p40 concentration in the culture supernatants were measured by ELISA. Data are presented as means ± SEMs of two or three independent experiments (n = 2 each). n.s., not significant, *$P < 0.05$ and ****$P < 0.0001$.

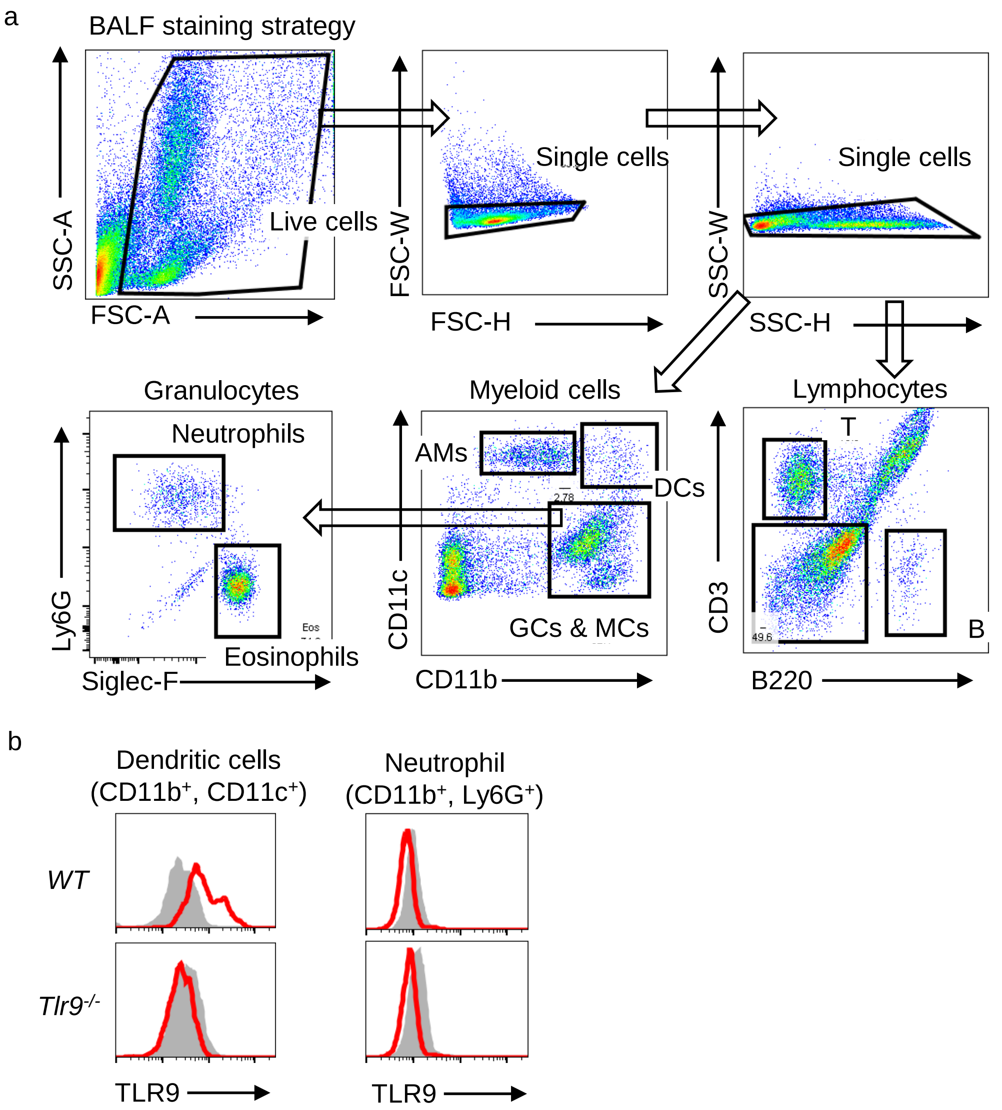

Figure S2. Flow cytometry analysis of bronchoalveolar lavage fluid (BALF) from house dust mite (HDM)-sensitized mice. (a) Protoc ol used for staining. BALF cells were stained for the markers indicated and analyzed. (b) Toll-like receptor 9 (TLR9) was detected in dendritic cells and neutrophils in BALF from wild-type (WT) and *Tlr9* $^{-/-}$ *mice* by using the anti-mouse-TLR9 monoclonal antibody NaR9. Gray histograms are isotype control. Red lines represent NaR9 staining. Data are presented from at least three independent experiments (n = 2 or 3 each). DCs: Dendritic cells, AMs: Alveolar macrophages, GCs: Granulocytes, MCs: Monocytes

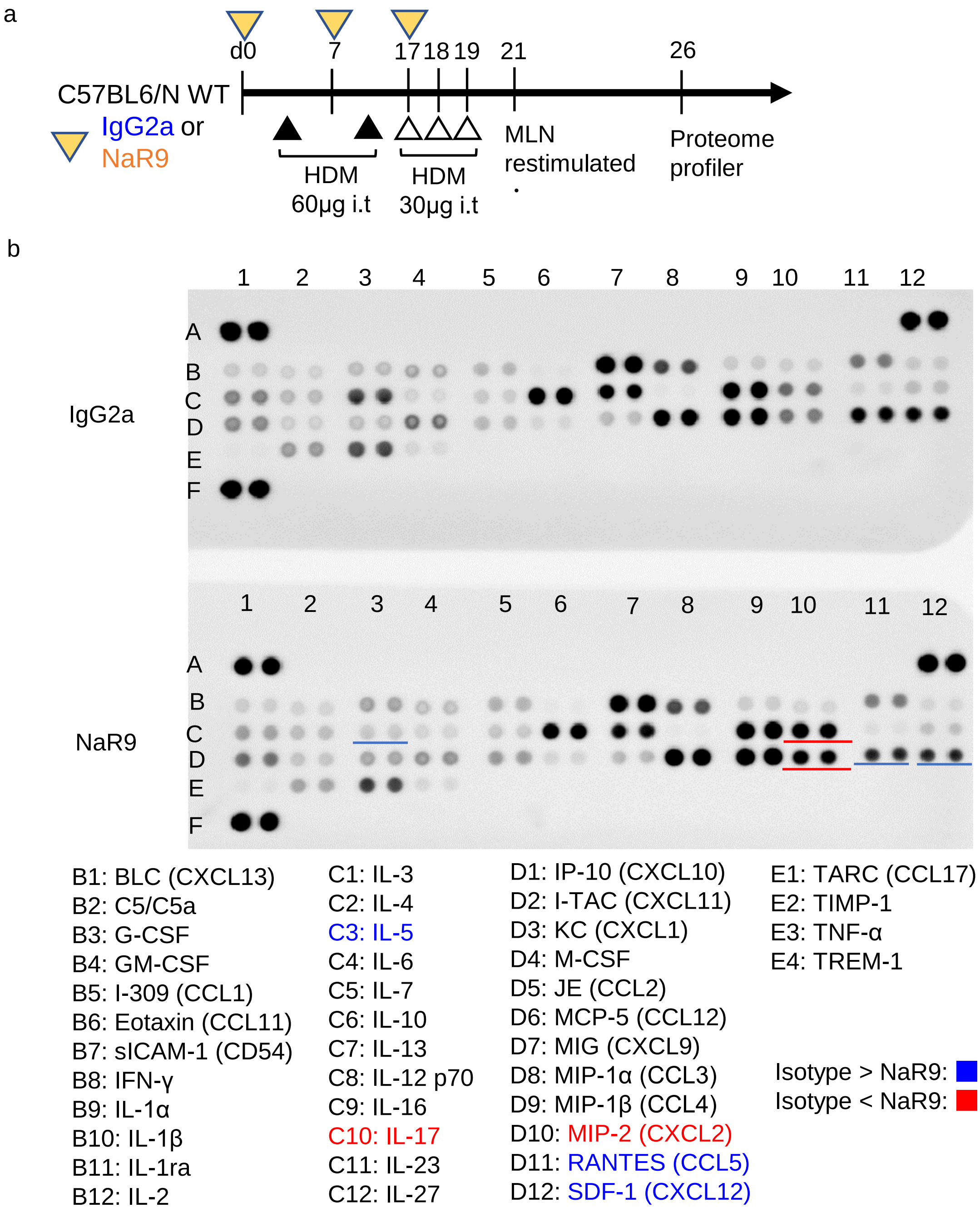

Figure S3. NaR9 affects the production of various cytokines in house dust mite (HDM)-induced allergic asthma. (a) Protocol used for HDM sensitization. (b) Mesenteric lymph node (MLN) cells were collected from HDM-sensitized mice treated with isotype control or the anti-mouse-TLR9 monoclonal antibody NaR9 and then restimulated with HDM. Five days later, the culture supernatants were analyzed with Proteome Profiler (R&D Systems). In b, red lines indicate upregulation upon NaR9 treatment. Blue lines indicate downregulation upon NaR9 treatment. i.t., intratracheally.

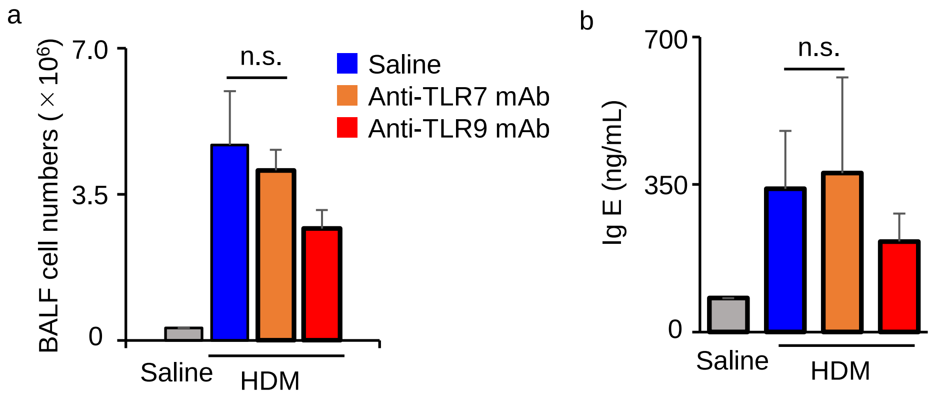

Figure S4. Anti-mouse-TLR7 monoclonal antibody (mAb) (A94B10) does not alter house dust mite (HDM)-induced allergic inflammation. (a) Total cell numbers were counted in the bronchoalveolar lavage fluid (BALF) of HDM-sensitized C57BL/6 mice treated with saline, anti-mTLR7 mAb, or anti-mTLR9 mAb. (b) Sera were collected from HDM-sensitized C57BL/6 mice treated with saline or the indicated mAbs and the IgE titers measured by ELISA. Data are presented as means ± SEMs of three independent experiments (n = 2 or 3 each). n.s., not significant.

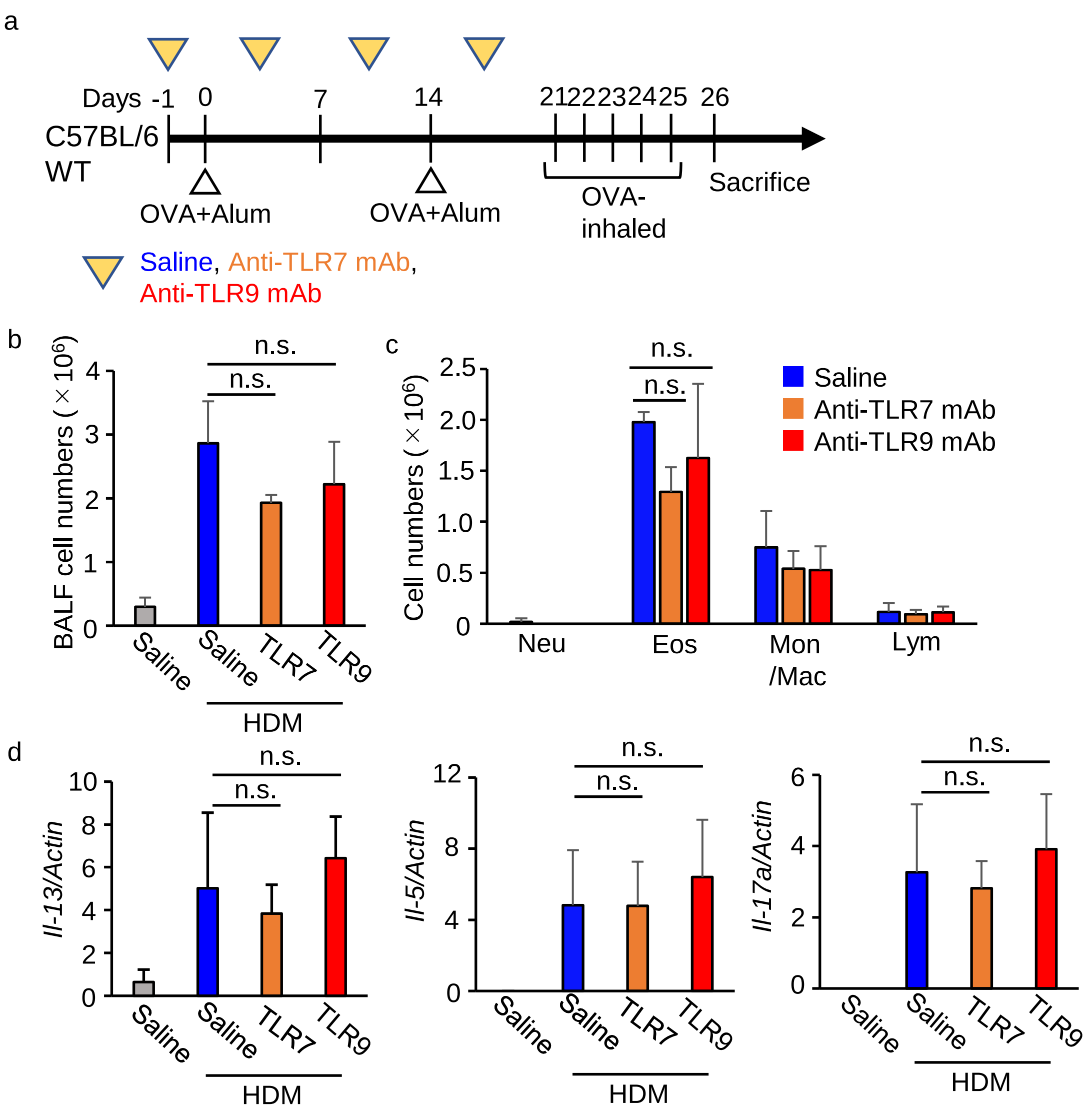

Figure 5S. Toll-like receptor (TLR)7 and TLR9 are not involved in allergic asthma induced by ovalbumin (OVA) and alum. (a) OVA–Alum sensitization strategy is shown. (b) Total cell numbers were counted in the bronchoalveolar lavage fluid (BALF) of OVA–alum-sensitized C57BL/6 mice treated with saline, anti-mTLR7 monoclonal antibody (mAb), or anti-mTLR9 mAb. (c) Neutrophils (Neu), eosinophils (Eos), monocytes/macrophages (Mon/Mac), and lymphocytes (Lym) were counted in the BALF after Giemsa staining. (d) Lung *Il-13, Il-5, Il-17a* and *β-actin* were measured in OVA–alum-sensitized C57BL/6 mice treated with saline or the indicated mAbs. Data are presented as means ± SEMs of three independent experiments (n = 4 or 5 each). n.s., not significant.
